# Nutritive Value Variation of Paunch Manure as an Alternative Feed Ingredient

**DOI:** 10.3390/ani11123573

**Published:** 2021-12-16

**Authors:** Taylor Jacob Garcia, Nichole Marie Cherry, Kimberly Ann Guay, Jeffrey Alan Brady, James Pierre Muir, William Brandon Smith

**Affiliations:** 1 Department of Animal Science, Tarleton State University, Stephenville, TX 76401, USA; taylor.garcia@go.tarleton.edu (T.J.G.); guay@tarleton.edu (K.A.G.); 2 Texas A&M AgriLife Research, Stephenville, TX 76402, USA; nichole.cherry@ag.tamu.edu (N.M.C.); jeff.brady@ag.tamu.edu (J.A.B.); jim.muir@ag.tamu.edu (J.P.M.); 3 Department of Wildlife and Natural Resources, Tarleton State University, Stephenville, TX 76401, USA

**Keywords:** in vitro digestibility, sustainable livestock production, novel feeds, sustainability, cattle

## Abstract

**Simple Summary:**

Sustainable livestock production is a benchmark for advancements in animal nutrition. Recovery of nutrients from products currently disposed of provides a potential solution to this mandate. Paunch manure is a potential novel feed ingredient comprised of digested feedstuffs at different stages of degradation, saliva, microorganisms, and products of fermentation. Thus, our objective was to determine the variability in nutritive values of dried paunch manure collected from harvested cattle. Animal-to-animal variation accounted for 30% to 75% of the variance in all measures of nutritive value. We believe that dried paunch manure may be a viable feed ingredient for inclusion in livestock rations, but a centralized composting system may be necessary to increase consistency.

**Abstract:**

Ruminants, which have multi-compartmented stomachs, are adapted to digest cellulosic materials, which constitute the primary expense on ranches and dairies. Industrial byproducts can be repurposed for livestock diets to decrease these costs. Therefore, finding alternative feedstuffs may benefit the economics of livestock production. The goal of this project was to evaluate the variation in nutritive value of ruminal waste as a potential feedstuff. Twelve paunch samples were collected from individual cattle across multiple harvest dates at the Tarleton State University Meat Laboratory, Stephenville, TX. Samples were dried and assayed for dry matter (DM), crude protein (CP), sequential neutral detergent fiber (NDF), acid detergent fiber (ADF), and acid detergent lignin (ADL), and physically-effective fiber (peNDF). Samples were subjected to batch-culture in vitro digestibility assays for the determination of digestibility coefficients. Mean NDF, ADF, ADL, CP and peNDF concentrations were 681, 399, 109, 150, and 387 g kg^−1^ DM, respectively. Contribution to variance from sample for NDF, ADF, ADL, CP, and peNDF were 75.3, 41.9, 33.0, 51.2, and 71.3%, respectively. In vitro true digestibility (IVTD) and in vitro NDF digestibility (IVNDFD) were recorded as 462 and 216 g kg^−1^ DM, respectively. Contribution to variation of sample for IVTD and IVNDFD were 31.0 and 30.7%, respectively. Results indicate that rumen waste harvested from abattoirs may be useful for sustainable livestock production, while reducing environmental threats posed by disposal, but the viability of the product is highly dependent on the source animal. For full viability of application in a sustainable system, a centralized receiving and compositing system may be useful for developing a consistent product.

## 1. Introduction

Meat and milk produced by ruminant animals are important agricultural sources of protein to humans. Ruminant production is of considerable economic value and sustains food security in many areas around the world. However, the sector faces major obstacles due to diminishing natural resources and increases in production costs [1]. Increasing costs associated with waste disposal are forcing abattoirs to reconsider present concepts for abattoir by-product management.

Ruminant animals develop a diverse and complex microbial ecosystem for digesting fibrous feedstuffs. The particle size of plant material is reduced through the action of mastication and rumination, and microbial action further reduces this material into smaller digestible components by bacteria and other microbes. Ruminal content, also known as paunch manure, represents a potential alternative cattle feed. Paunch manure consists of fermented and non-fermented dietary feed that passed various digestion stages [2], including microbial metabolic end products such as microbial protein, amino acids, vitamins, and volatile fatty acids without any anti-nutritional factors [3]. Recovering these nutrients provides a potential solution to waste disposal and pollution mitigation, as well as reducing feed costs associated with producing livestock [4].

The chemical composition of dried paunch manure (DPM) has been previously characterized in relation to non-ruminant feeding [5]. Refs. [3,6] reported that DPM CP content ranged from 90 to 200 g kg^−1^ DM and could be a viable protein source for livestock if properly processed. Successful implementation of DPM into diets of Nile Tilapia (*Oreochromis niloticus*) [7], African catfish (*Clarias gariepinus*) [8], broilers (*Gallus gallus domesticus*) [6], layers (*Gallus gallus domesticus*) [9], and rabbits (*Oryctolagus cuniculus*) [3] have been recorded. Paunch-manure nutrient levels depend primarily on the ration fed to the animal before harvest [10]. However, in the practical application of this potential feeding system, prior diet would be an unknown factor; thus, this would be viewed as variation in animal source. An understanding of the variability in nutrient content would provide practitioners with the necessary information to make use of this waste product. The objectives of our experiment were two-fold: (1) what is the nutritive value of DPM, and (2) what is the variation in characteristics of DPM. Each of these objectives was designed to assess the suitability of DPM as a potential novel feed ingredient.

## 2. Materials and Methods

### 2.1. Experimental Design and Sample Collection

Our experiment was conducted as a completely randomized design. Rumen paunch samples were collected from twelve cattle (*Bos taurus taurus*, *B. taurus indicus*, or *B. taurus indicus* × *B. taurus taurus*) selected at random from those harvested at the Tarleton Meat Laboratory, Stephenville, TX, USA. Samples were collected on four different days: four on 29 March, four on 20 April, one on 4 May, and three on 11 May 2018. As common in abattoirs, animals had been fasted overnight prior to harvest. Paunch samples collected served as the experimental unit. Paunch manure was collected fresh immediately from the abattoir offal room. Digestive organs were removed from each harvested animal, placed in an offal barrel, and punctured with a knife. Rumen content representing the solid material was collected from different locations within the rumen and pooled. Samples were transported to the laboratory at Texas A&M AgriLife Research and Extension Center at Stephenville, TX, USA (1.6 km). Harvested cattle feed history was not available and, thus, was considered as part of the random variation among cattle; this was an intentional part of the project to represent a practical setting. Approximately 1.5 kg of solid paunch manure was collected from each rumen. Rumen liquor was removed by straining samples through eight layers of cheesecloth.

### 2.2. Nutritive Value Assays

Harvested samples were dried in a forced air gravity convection oven to a constant weight at 55 °C until weight loss ceased (approximately 72 h). Samples were ground to pass through a 2-mm screen using a Wiley mill (Arthur H. Thomas Co., Philadelphia, PA, USA), and a subsample was ground to pass through a 1-mm screen. Neutral detergent fiber (NDF) and acid detergent fiber (ADF) were measured sequentially using the ANKOM^200^ Fiber Analyzer (ANKOM Technology Corporation, Fairport, NY, USA) [11]. The procedure included sodium sulfite and α-amylase, and values were expressed inclusive of residual ash. Acid detergent lignin (ADL) was determined using the sulfuric acid method (Method 973.18) [12]. Nitrogen was measured using the Dumas total combustion method (Elementar Americas, Mt. Laurel, NJ, USA; Method 990.09) [12] and CP was calculated based on the nitrogen content of each sample (N × 6.25).

Physically-effective fiber (peNDF) content was calculated using unground solid paunch material. Particle size distributions were determined using the manually-operated Penn State Particle Separator (Nasco, Fort Atkinson, WI, USA) containing three sieves (19, 8, and 4 mm) and a pan. The material remaining on each sieve and pan was weighed (scale accurate to 1 g). The operation of the device was similar to that described in [13]. The physical effectiveness factor was calculated as the sum of the proportions of the materials retained on the 19.0- and 8.0-mm sieves. The peNDF was determined by multiplying the physical effectiveness factor by the total NDF content of the sample [14].

In vitro true digestibility (IVTD) was determined by the DAISY^II^ Incubator (ANKOM Technology Corporation, Fairport, NY, USA). Rumen inoculum was collected fresh from an Angus steer (approximately 8 year old; 544 kg) fitted with a rumen cannula. ANKOM F57 filter bags (ANKOM Technology Corporation, Fairport, NY, USA) were pre-rinsed in acetone for 5 min and completely air-dried. Weight from each F57 filter bag was recorded. Representative 0.5-g samples, ground through a 2-mm screen, were placed into filter bags and heat-sealed. Heat-sealed bags were placed in the DAISY^II^ Incubator (ANKOM Technology Corporation, Fairport, NY, USA). Each incubation jar contained paunch samples from four different sources, with four replications, as well as two blank bags. The blank bag was used to calculate a correction factor that adjusted for weight loss or gain from the filter bags. Samples were incubated for 48 h in a rumen fluid/buffer solution [15].

After incubation, jars were removed and fluid drained. Samples were removed, rinsed with water, boiled in an NDF solution using an ANKOM^200^ Fiber Analyzer to remove microbial debris and any remaining soluble fractions, and dried at 105 °C for 24 h. Post-in vitro NDF weight was recorded. In vitro true digestibility [16] was calculated as

%IVTD= 100 − (W_3_ − (W_1_ × C_1_)) × 100/(W_2_ × DM) (1)

where W_1_ = bag tare weight, W_2_ = sample weight, W_3_ = final bag weight after in vitro and NDF treatment, C_1_ = blank bag correction (final oven-dried weight/original weight). In vitro NDF digestibility (IVNDFD) was calculated with the same equation where W_2_ was multiplied by NDF, rather than DM, weight.

### 2.3. Statistical Analysis

Data were analyzed as a random-effects model using PROC MIXED in SAS^®^ v. 9.4 (SAS Institute, Cary, NC, USA). A random-effects model is a statistical model in which no fixed effects are present and random effects are included to estimate the contribution to model variance. The COVTEST option was used to obtain *p*-values for random effects. The model statement included no effects, and denominator degrees of freedom were adjusted for a small sample size using the Kenward-Roger approximation [17]. The random statement included the effect of the sample (individual animal) and harvest date (a stand-in for source and diet). The model equation was described as

y_ij_ = μ + S_i_ + D_j_ + ε_ij_ (2)

where y_ij_ is the dependent variable, μ is the overall mean, S_i_ is the random effect of sample, D_j_ is the random effect of harvest date, and ε_ij_ is the random residual error. Response variables included NDF, ADF, ADL, and CP. For response variables IVTD and IVNDFD, the random effect of rep within the sample was also included to capture laboratory variation.

Multiple α levels were defined for this experiment. The first was defined as α_1_ = 0.05, such that differences among responses would be declared when *p* < 0.05. The second was defined as α_2_ = 0.10, such that tendencies for differences among responses would be declared when 0.05 ≤ *p* < 0.10.

## 3. Results

Mean, minimum, and maximum values, as well as variance components of random effects from NDF, ADF, ADL, CP, peNDF, IVTD, and IVNDFD are presented in Table 1. In an evaluation of variance components, sample (our stand-in for animal variation) was the only random effect that met the threshold for statistical significance (either α_1_ or α_2_). That said, in the case of ADF, ADL, IVTD, and IVNDFD, harvest date (our stand-in for animal source and diet) accounted for more variance than did sample; however, in each of these cases, the standard error of the effect was larger than the observed effect.

The particle size distribution of the DPM is presented in Table 2. Similar to the observations from nutritive value (chemical) components, the only significant source of variance for particle size (physical) was the sample. In contrast to the nutritive value components, however, the estimate for a variance from harvest date exceeded the estimate from the sample in only one instance (>19-mm). It should also be noted that the contribution to variance from the sample increased as particle size decreased. Another interesting observation from these data is that the combination of sample and harvest date accounted for at least 96% of the overall variance for each of the particle size distribution measures, meaning that there are few extraneous, or unaccountable, sources of variation in these data.

## 4. Discussion

### 4.1. Nutritive Value

Fiber concentrations of DPM from our experiment were near the upper limit of those values reported in the literature (Table 3). Neutral detergent fiber values previously reported ranged from 424 to 787 g kg^−1^ DM, while ADF concentrations have been reported to range from 209 to 545 g kg^−1^ DM [6,18,19,20,21]. Acid detergent lignin was not reported in this literature; however, ADL constituted 109 g kg^−1^ DM in our DPM samples. Concentrations of CP in DPM from our experiment were consistent with previous reports; however, [3] and [6] reported CP in DPM ranged from 136 to 153 g kg^−1^ DM, compared with 119 to 225 g kg^−1^ DM in the current study. In contrast, [22] found DPM pellets to have CP concentrations of 407 g kg^−1^ DM, though this was only after the addition of 100 g urea kg^−1^ DM.

A discussion of the suitability of DPM as a novel feed ingredient must also take into account the diet of the animal prior to harvest. Ref. [20] noted that nutritive value parameters, especially CP, can vary widely based on the pre-harvest diet. From the DPM samples collected, noticeable differences in hue, odor, and consistency were noted, but not quantified. Notably, inside the rumen of one of the animals harvested, there was visual evidence of concentrate feed (i.e., corn kernels) found within the fiber mat. Accurate feed history of cattle prior to slaughter could represent an avenue by which the dietary effect on the nutritive value of DPM may be examined. These values were probably influenced by pre-slaughter feeding regimen, length of time animals were fasted prior to slaughter, seasonality, feed resource diversity, and selectivity of pasture by different animals in different locations. It should also be noted, though, that if DPM were to become a viable feed ingredient, a pre-harvest diet would not be known, and contents from multiple animals would likely be pooled in a bulk tank at the abattoir. While pre-harvest diet is of great concern to the nutritive value of the material, and it is of academic concern in these evaluations, it is of little practical relevance in the application of this research.

### 4.2. In Vitro Digestibility

Both IVTD and IVNDFD digestive values were dependent upon the sample (Table 1). As for the chemical composition, this was probably due to the feeding regimen prior to slaughter, and how long the animals were restricted from the feed. The methodology of measuring in vitro digestibility, though, may impact the values obtained. Ref. [24] showed that there was no difference in IVTD when different sources of inoculum were used for feedstuffs varying in NDF and CP content. Ref. [25], however, found that the type of diet fed to the donor animal did affect the values of IVTD.

### 4.3. Particle Size Distribution

Ref. [26] demonstrated a physical form classification system used to describe feeds directly from particle size. According to this classification system, particles greater than 19 mm would be classified as long (e.g., grass hay), coarsely chopped (48 to 80 mm), or medium-coarse chopped (24 to 40 mm) while particles less than 19 mm but greater than 8 mm would be classified as medium-chopped. Particles less than 8 mm but greater than 1.18 mm would be classified as medium-finely chopped, and anything less than 1.18 mm would be classified as finely-chopped or ground. By using this classification system, 206 g DPM kg^−1^ DM were coarsely-chopped or medium-coarse chopped, 361 g kg^−1^ were medium-chopped, 186 g kg^−1^ were finely-chopped, and 247 g kg^−1^ were ground.

Ref. [27] recommended distributions of particle size for corn silage, haylage, and TMR (Table 4). Particle retention on the 19-mm sieve of DPM in our study was greater than the optimum recommended for corn silage and TMR, but only slightly greater than the haylage recommendation; however, one DPM sample did fall in the range for corn silage and TMR (31 g kg^−1^ DM). The increase on the 19-mm sieve reduced retention on the 8-mm sieve. Values were within the range of corn silage, haylage, and TMR guides for medium particles. The proportion retained on the 4-mm sieve was slightly lower than the range for haylage. The particle size of the DPM collected may have been skewed in the drying process because, prior to dryer entry, rumen content was manually strained to rid liquid fraction. This may have led to a loss of smaller particles. It is also possible that the skewness of the particle size data may have arisen from the drying of the material since most applications of the Penn State Particle Separator use wet or air-dried material.

Ref. [28] does not specify requirements for physically effective fiber. However, the physical makeup of the diet, including forage chop, are critical considerations when developing a ration, particularly relating to reducing the risk of acidosis [29]. The resistance to flowing particles that exit the rumen is heavily governed by particle size. It has been shown that, in sheep, only a very small proportion (10 to 30 g kg^−1^) of particles greater than 1.18-mm passed out of the reticulorumen [30]. This observation has given rise to the size theory, stating that most feed particles must be reduced to at least 1.18-mm before exiting the rumen for further digestion. This suggested threshold in lactating dairy cows is theorized to be greater than 1.18-mm and may be accurately described to be between 3 to 5 mm [31,32]. Regardless, [26] proposed multiplying the NDF content of a feed by the proportion of material remaining on the 1.18-mm sieve as a potential measure of peNDF that simulates chewing activity and contributed to the formation of the fiber mat. Given the increased use of the Penn State Particle Separator on commercial farms, 19- and 8-mm sieves have been proposed to replace the use of the 1.18-mm sieve when measuring peNDF [33].

### 4.4. Other Considerations

A question surrounding the use of DPM pertains to the availability of the material. Ref. [23] stated that, at the time of harvest, a steer contains approximately 3.5 kg of DPM. This number, however, seems low and is provided without reference to the body size of the animal. Ref. [34] found that DM fill of mature cows ranged from 8.7 to 17.0 g DM kg^−1^ BW (3.9 to 7.7 kg for a standard bovine animal unit [454 kg]). By comparison, a standard bovine animal unit should consume 9 kg of DM daily, assuming 2% BW in DMI. This means that it would take three harvested steers to provide the necessary DM to feed one animal unit per day. For the application of these data, it would take multiple large abattoirs to start a collection for this to be a viable system.

Another concern related to DPM as a novel feed ingredient is safety. Ref. [35] claimed that dried rumen content not only contained microbial protein and other nutrients, but also cattle salivary secretions. This represents a potential limitation to the use of dried rumen content from slaughterhouses as a dietary additive, given that DPM could pass along transmissible diseases. Heat application may overcome this potential for disease transmission [36], but the excess application of heat may also result in decreased feed value, thus negating the original purpose.

## 5. Conclusions

Results indicate that harvesting rumen waste from slaughterhouses may be a useful ingredient for sustainable livestock production while reducing the environmental threat posed by disposal of ruminal waste, but that the viability of the product is highly dependent on the source animal. Caution should be used in making broad assumptions from these data, however, given the small sample size. For full viability of application in a sustainable system, a centralized receiving and compositing system may be useful to develop a consistent product.

Much remains to be investigated with regard to the definitive recommendation of dried paunch manure as a feed ingredient. For future studies, we would recommend investigation into (a) the pathogen load of DPM and methods for decreasing these loads, if present; (b) full characterization of the chemical fractions of DPM, especially CP, and different digestibility rates of each, and (c) feeding experiments with larger animals, primarily ruminants, to compare with the small, nonruminant studies that exist in the literature.

## Figures and Tables

**Table 1:** Nutritive value and in vitro digestibility of dried paunch manure obtained from cattle harvested at the Tarleton Meats Laboratory, Stephenville, TX, USA.

Component ^1^, g kg^−1^ DM	Mean	Minimum	Maximum	Variance Components, %		
				Sample	Harvest Date	Residual
NDF	681	555	755	75.3 **	20.8	3.8
ADF	399	291	496	41.9 **	54.7	3.4
ADL	109	33	175	33.0 *	52.9	14.1
CP	150	119	225	51.2 **	35.4	13.4
peNDF	387	262	474	71.3 **	24.1	4.6
IVTD	462	270	692	31.0 **	68.2	0.5
IVNDFD	216	10	543	30.7 **	68.2	1.0

^1^ NDF = neutral detergent fiber, assay with α-amylase and sodium sulfite and expressed inclusive of residual ash; ADF = acid detergent fiber, expressed inclusive of residual ash; ADL = acid detergent lignin; CP = crude protein (N × 6.25); peNDF = physically-effect NDF; IVTD = in vitro true digestibility; IVNDFD = in vitro NDF digestibility. * 0.05 ≤ *p* < 0.10. ** *p* < 0.05.

**Table 2:** Particle size distribution of dried paunch manure obtained from cattle harvested at the Tarleton Meats Laboratory, Stephenville, TX, USA.

Sieve Pore Size ^1^, mm	Physically-Effective Fiber, g kg^−1^ DM			Variance Components, %		
	Mean	Minimum	Maximum	Sample	Harvest Date	Residual
19.0	206	31	410	49.2 **	49.8	1.0
8.0	361	258	451	50.7 **	47.0	2.3
4.0	186	146	305	89.1 **	6.2	4.7
Pan	247	186	357	96.5 **	0.0	3.5

^1^ Pore size represents each layer of the Penn State Particle Separator. ** *p* < 0.05.

**Table 3:** A comparison of nutritive value of dried paunch manure from previously published literature.

Species	NDF ^1^	ADF ^2^	CP ^3^	Source
Cattle	681	399	150	current experiment
Cattle	-	-	185	[6]
Cattle	-	-	186	[7]
Cattle	-	-	218	[18]
Cattle	592	367	142	[19]
Camel	653	399	110	[19]
Sheep	516	319	150	[19]
Cattle	424	209	194	[20]
Cattle	787	545	126	[21]
Sheep	-	317	140	[23]

^1^ NDF = neutral detergent fiber, assayed with α-amylase and sodium sulfite and expressed inclusive of residual ash. ^2^ ADF = acid detergent fiber, expressed inclusive of residual ash. ^3^ CP = crude protein (N × 6.25).

**Table 4:** Particle size distribution, g kg^−1^, of dried paunch manure compared with recommendations for corn silage, haylage, and total mixed rations (adapted from [27]).

Sieve Pore Size ^1^, mm	DPM ^2^	Recommendations		
		CS ^3^	Haylage	TMR ^4^
19.0	206	30 to 80	100 to 200	20 to 80
8.0	361	450 to 650	450 to 750	300 to 500
4.0	186	300 to 400	200 to 300	300 to 500
Pan	247	Less than 50	Less than 50	Less than 50

^1^ Pore size represents each layer of the Penn State Particle Separator. ^2^ DPM = dried paunch manure; values are from the current experiment. ^3^ CS = corn silage. ^4^ TMR = total mixed ration.

## Data Availability

The data presented in this study are available on request from the corresponding author.
